# Laparoscopic disconnection of the hernial sac: is it enough for the treatment of congenital inguinal hernia in children?

**DOI:** 10.1007/s10029-025-03471-z

**Published:** 2025-10-13

**Authors:** Sameh Shehata, Israa Saad, Mohamed Abouheba, Mostafa Zain

**Affiliations:** Pediatric Surgery Department, Faculty of Medicine, Alexandria University, Alexandria, Egypt

**Keywords:** Inguinal hernia, Laparoscopy, Sac disconnection, Sutureless hernia repair

## Abstract

**Background:**

Laparoscopic repair of congenital inguinal hernia (CIH) has evolved with various techniques. Some advocate for sac disconnection alone, while others emphasize additional peritoneal closure or internal ring narrowing to prevent recurrence. This study evaluates the efficacy of laparoscopic sac disconnection alone versus disconnection with iliopubic tract repair (IPTR) for CIH, stratified by internal ring diameter (IRD), analyzing recurrence rates and postoperative complications.

**Methods:**

This study (April 2021- December 2023) included 208 children with CIH in the age range from 3 months to 12 years. Patients were stratified by IRD: Group A (≤ 1 cm, *n* = 142) underwent sac disconnection alone; Group B (> 1 cm, *n* = 66) received additional IPTR. Outcomes assessed included recurrence rates, operative time, and postoperative complications.

**Results:**

No hernia recurrences were observed in either group during the one-year follow-up. Group A had significantly shorter operative times (mean: 21.17 ± 5.42 min for unilateral cases) compared to Group B (37.61 ± 5.79 min). Postoperative scrotal edema occurred in 1.4% of Group A patients and 12.1% of Group B patients, but all cases were resolved spontaneously. No intraoperative injuries to the vas deferens or testicular vessels were reported.

**Conclusion:**

Laparoscopic sac disconnection alone is sufficient for CIH with IRD ≤ 1 cm, offering shorter operative times with excellent outcomes. For larger defects (> 1 cm), IPTR provides durable repair despite longer operative times. This IRD-based algorithm optimizes outcomes while maintaining procedural simplicity.

**Supplementary Information:**

The online version contains supplementary material available at 10.1007/s10029-025-03471-z.

## Introduction

Laparoscopic inguinal hernia repair emerged in the 1990 s as a minimally invasive alternative to traditional open surgery, revolutionizing the management of congenital inguinal hernias (CIH) [1]. This approach was initially met with skepticism due to concerns about technical complexity and the learning curve associated with laparoscopic techniques. However, over time, it has gained widespread acceptance and is now considered the standard approach in many centers worldwide [2, 3]. Since its first introduction and with increasing surgical expertise, numerous technical modifications have been introduced, which can be categorized into three distinct conceptual approaches.

The first approach involves ligation of the hernial sac without disconnection, wherein sutures are placed to occlude the patent processus vaginalis (PPV) while keeping its continuity. This can be achieved through intracorporeal techniques, such as Montupet’s purse-string suture [4] or Schier’s “N”-shaped suture technique [5], or extracorporeal methods, as described by Patkowski “PIRS Technique” [6]. A further modification of this technique involves cauterization of the peritoneum around the internal ring (IR) before closure to create a scar, which enhances the strength of the repair “Burnia” [7]. The second approach, as proposed by Becmeur et al. [8] entails disconnection of the hernial sac with subsequent closure of the peritoneal defect. The third approach, described by Riquelme et al. [9], involves complete disconnection and resection of the hernial sac along with the parietal peritoneum surrounding the IR. In this technique, Repair of the deep ring is performed selectively, only when the ring diameter exceeds 10 mm.

These diverse approaches aimed to simplify the procedure, enhance its feasibility, and minimize both complications and recurrence rates. However, there is currently no definitive evidence to support the superiority of one technique over others [2, 7].

The aim of this study was to assess the concept of peritoneal disconnection alone without peritoneal closure in the management of inguinal hernia in children. When the deep ring is wider than 10 mm, repair of the wide deep ring will be done, again without peritoneal closure.

## Materials and methods

This study included children in the age range from 3 months to 12 years who underwent laparoscopic repair of CIH at our center from April 2021 to December 2023. Patients with the following conditions were excluded: incarcerated CIH, recurrent CIH, associated undescended testis, associated hydrocele, and chronic comorbidity such as congenital heart disease, severe pulmonary disorders, or ventriculoperitoneal shunt.

The patients were divided into two groups according to the intraoperative findings. The first group (A) included patients with a transverse internal ring diameter (IRD) of 1 cm or less who underwent laparoscopic disconnection of the hernia sac without peritoneal closure or internal ring narrowing. The other group (B) included patients with a transverse IRD measuring at more than 1 cm who underwent laparoscopic disconnection of the hernia sac and iliopubic tract repair (IPTR). The main objective of the study was to determine the recurrence rate within a year, in addition to comparing the two groups in terms of operative time, efficacy, safety, and postoperative complications.

### Operative technique

The patients were placed in a supine position. The surgeon stood at the head of the table with the assistant on his left side, the scrub nurse on his right side, and the laparoscopic monitor at the feet of the table. A transumbilical 5 mm port was inserted using an open technique to establish pneumoperitoneum. Insufflation of the abdomen is initiated with a flow of 1.5 L/min at a pressure of 4–6 mmHg, then the pressure was raised to 8–12 mmHg according to the patient’s weight. Then, a 5-mm 30°-endoscope was introduced. Laparoscopic exploration was done to inspect the abdominal cavity and identify the IR on both sides to confirm the clinical diagnosis and manually reduce any hernial content from outside. Two 3- or 5-mm portless instruments were inserted at the right and the left mid-clavicular lines at the level of the umbilicus to maintain triangular orientation. The patient was put in Trendelenburg’s position to withdraw the abdominal viscera away from the IR.

**Fig. 1:**
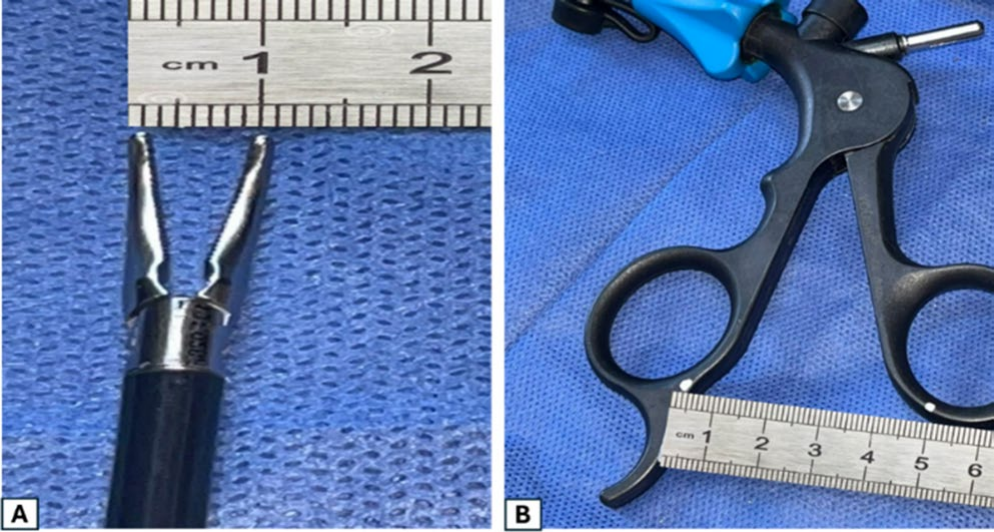
With the jaw of Maryland opened to measure 1 cm (**A**), two points were marked on both hands of Maryland with the distance in between adjusted to be 5 cm (**B**)

**Fig. 2:**
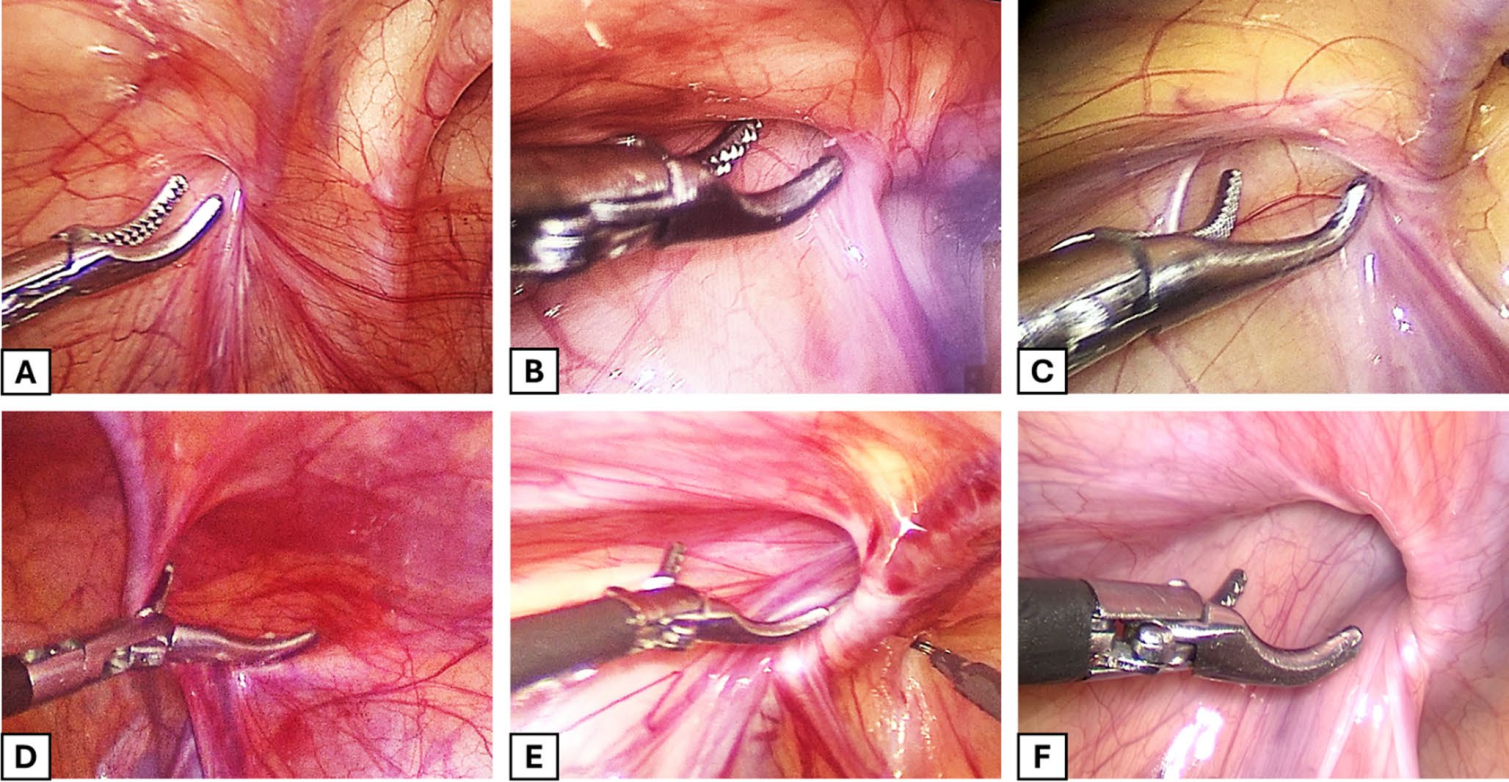
Measurement of the transverse diameter of the internal ring. **(A-C)** Examples of cases with transverse diameter < 1 cm. **(D-F)** Examples of cases with transverse diameter > 1 cm.

The transverse IRD was measured using the jaw of the Maryland. Two points were marked on both hands of the Maryland with a distance in between adjusted to be 5 cm, while the jaw of the Maryland is opened to measure 1 cm (Fig. 1). The jaw of Maryland is opened against the transverse diameter of the IR, and the distance between the two marks was measured with a ruler. If the distance between the marked points was ≤ 5 cm, the transverse diameter of the IR was ≤ 1 cm, and the patient was assigned to group (A). If it measured more than 5 cm, the transverse diameter of the IR was > 1 cm, and the patient was assigned to group (B). Fig. 2.

In both groups, dissection was started using a Maryland and a hook diathermy to create two small rents in the peritoneum on both sides of the vas and vessels. The edge of the peritoneal rent was held with Maryland, and the vas deferens and testicular vessels were swept away from the peritoneum with the knee of the hook without using the diathermy. Then, the resultant transparent peritoneal band over the vas deferens and testicular vessels was divided. After that, the dissection was continued circumferentially with the monopolar hook electrocautery until the peritoneum at the IR was completely disconnected and the distal part of the sac dropped into the inguinal canal.

**Fig. 3:**
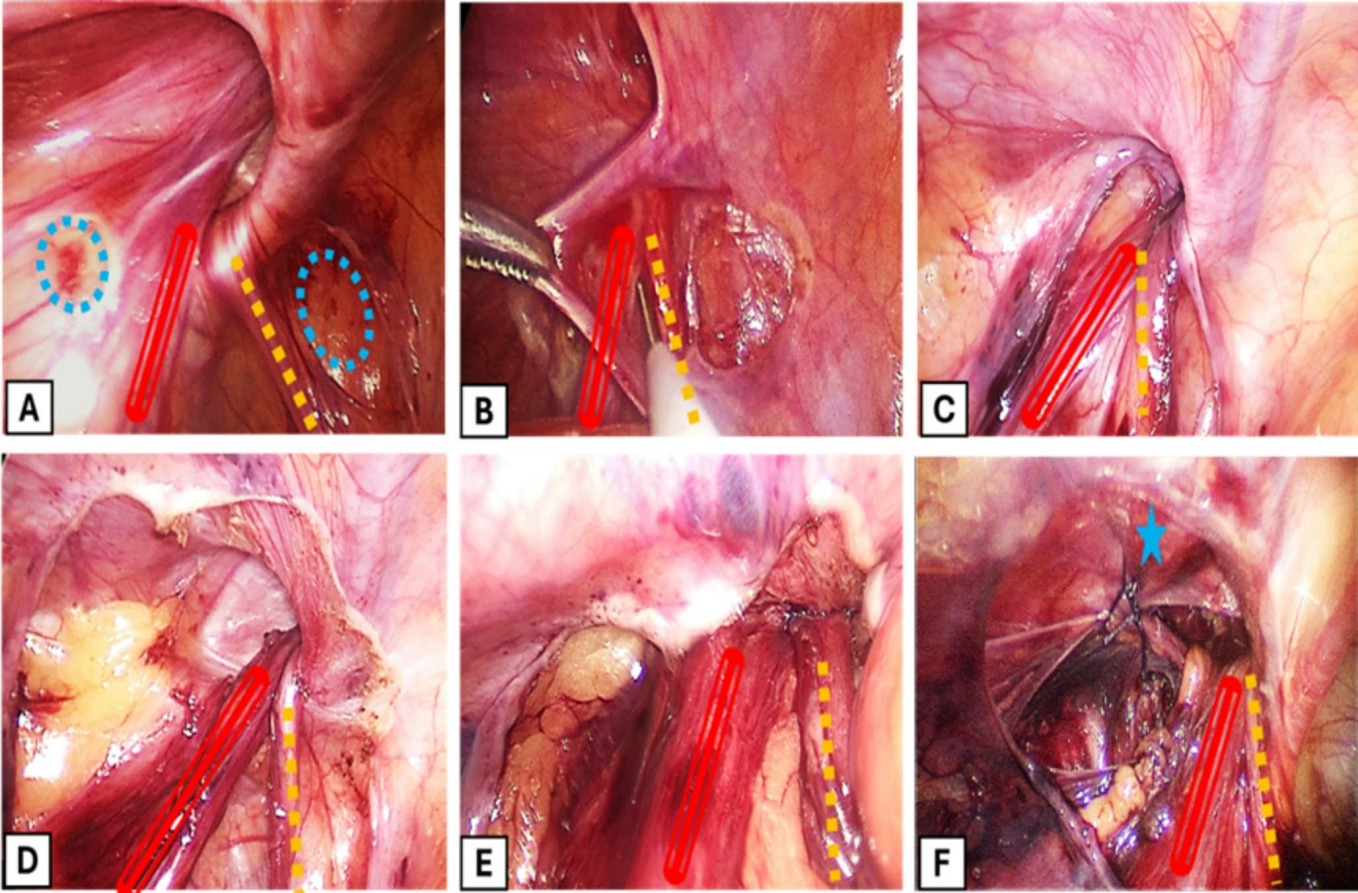
Steps of the procedure. (**A**) Two small rents in the peritoneum were created on both sides of the vas and vessels using a hook electrocautery (blue dashed circles). (**B**) Elevation of the edge of the peritoneal rent with the Maryland and sweeping away the vas deferens and testicular vessels with the knee of the hook. (**C**) The resultant transparent peritoneal band over the vas deferens and testicular vessels was divided. (**D**) Complete disconnection of the hernial sac. (**E**) The view after release of the pneumoperitoneum shows complete collapse of the internal ring. (**F**) In cases with transverse diameter >1 cm, narrowing the internal ring with one suture (blue star) approximating the iliopubic tract to the transverse arch of fascia transversalis (TATA). Parallel red lines: testicular vessels. Yellow dashed line: vas deferens

For group A, the procedure was completed at this step, while in group B, an additional step for narrowing the IR with Iliopubic tract repair (IPTR) was done. A 3/0 or 4/0 suture (Vicryl) on a half-circle needle was introduced with the needle holder through the portless incision. The iliopubic tract (IPT) was identified as a shiny white band running under the cord structures at the inferior border of the IR, while the transverse arch of fascia transversalis (TATA) was identified as the arching of the transversalis fascia immediately above and lateral to the IR. The IPT was approximated to TATA using an interrupted suture just lateral to the cord structures. Only one or two sutures were placed to avoid excessive tension, as this was usually sufficient. The needle was retrieved under vision through the stab incision. Finally, the fascia was closed with Vicryl sutures, and the skin was closed with Steri-Strips. Fig. 3.

Postoperatively, analgesics were given in the form of paracetamol and/or diclofenac suppositories, based on age. Oral feeding started 2 h after surgery. Patients were discharged on the same day. Patients were followed up in the outpatient clinic at 1 week, 1 month, 3 months, 6 months, and 1 year (or contacted by phone for the late 3, 6 months, and 1-year follow-ups, depending on patient accessibility and parental preference). The follow-up was to look for recurrence and any other complications.

## Statistical analysis of the data

The data were analyzed using the IBM SPSS Statistics software package, version 20.0 (©IBM Corp., Armonk, NY). The normality of continuous variables was assessed using the Shapiro-Wilk test. As the data were not normally distributed, non-parametric tests (Mann-Whitney U) were used to compare continuous variables. Where appropriate, categorical variables were analyzed using the Chi-square test or Fisher’s Exact Test. Monte Carlo simulation was applied when expected cell counts were low in multi-category comparisons. A p-value < 0.05 was considered statistically significant.

## Results

A total of 208 patients diagnosed with CIHs were included in the study and categorized into two groups based on intraoperative findings. Group A comprised 142 patients (68.3%) with a transverse internal ring diameter of 1 cm or less, while Group B included 66 patients (31.7%) with an internal ring diameter larger than 1 cm.

In Group A, 117 patients (82.4%) were male, and 25 (17.6%) were female, whereas Group B included 53 males (80.3%) and 13 females (19.7%). The difference in sex distribution between the two groups was not statistically significant (*p* = 0.687), ensuring comparability.

The age of patients at the time of surgery ranged from 0.25 years (3 months) to 12 years, with a mean of 5.89 ± 4.05 years in Group A, and from 0.33 years (4 months) to 11 years, with a mean of 5.73 ± 3.91 years in Group B. There was no significant difference in age distribution between the groups (*p* = 0.791). This finding suggests that age was not a confounding factor in the observed outcomes.

Regarding hernia laterality, in Group A, right-sided inguinal hernias were observed in 67 patients (47.2%), left-sided in 42 patients (29.6%), and bilateral in 33 patients (23.2%). In Group B, 37 patients (56.1%) had right-sided hernias, 21 patients (31.8%) had left-sided hernias, and 8 patients (12.1%) had bilateral hernias. The difference in hernia laterality was not statistically significant (*p* = 0.164).

A synchronous contralateral PPV was detected intraoperatively in 11 patients (5.3%), including 7 males and 4 females. In these cases, the IRD was less than 1 cm. Additionally, 12 patients (5.8%) had previously undergone an open herniotomy on one side and later developed a metachronous hernia on the opposite side.

The operative time (OT), measured from skin incision to skin closure, varied significantly between the two groups. In Group A, the OT for unilateral cases ranged from 15 to 30 min, with a mean duration of 21.17 ± 5.42 min, while bilateral cases had a mean OT of 37.32 ± 4.44 min. In Group B, the OT for unilateral cases ranged from 30 to 50 min, with a mean duration of 37.61 ± 5.79 min, while bilateral cases had a mean OT of 57.41 ± 3.81 min. The increase in operative time in Group B was statistically significant for both unilateral (*p* < 0.001) and bilateral procedures (*p* < 0.001), attributed to the additional step of internal ring narrowing.

Intraoperatively, two cases (0.96%) of minor periperitoneal bleeding were recorded—one in each group. In Group A, bleeding occurred during peritoneal incision, while in Group B, it resulted from needle insertion into the abdominal cavity. Both cases stopped spontaneously without intervention. No injuries to the vas deferens, testicular vessels, or retained needles were reported.

All patients recovered uneventfully, resumed oral feeding within two hours postoperatively, and were discharged on the same day.

All patients (100%) attended the 1-week and 1-month visits in person. At 3 months and 6 months, follow-up was successfully completed for all patients, with telephone assessments accounting for 28% and 45% of visits, respectively. At 1 year, 95% of patients were successfully contacted, with 72% of these assessments conducted via telephone.

Transient inguinal edema was observed in 2 patients (1.4%) in Group A and 8 patients (12.1%) in Group B. The difference was statistically significant (*p* = 0.002), indicating a higher incidence of inguinal edema in patients undergoing internal ring narrowing. However, the edema resolved spontaneously in all cases within three weeks. No cases of persistent inguinal edema, hematoma, hernia recurrence, or testicular atrophy were reported in either group throughout the one-year follow-up period. Table 1.

**Table 1:** Comparison between the two studied groups

Variable	Total (*n* = 208)	Group A (*n* = 142)	Group B (*n* = 66)	Statistical Test	*p*-value
Sex					
Male	170 (81.7%)	117 (82.4%)	53 (80.3%)	χ² = 0.16	0.687
Female	38 (18.3%)	25 (17.6%)	13 (19.7%)		
Age (years)					
Min. – Max. Mean ± SD.	0.25–12 (5.85 ± 3.95)	0.25–12 (5.89 ± 4.05)	0.33–11 (5.73 ± 3.91)	U = 10,741	0.791
Hernia Side					
Right	104 (50.0%)	67 (47.2%)	37 (56.1%)	χ² = 3.61 (MC)	0.164
Left	63 (30.2%)	42 (29.6%)	21 (31.8%)		
Bilateral	41 (19.8%)	33 (23.2%)	8 (12.1%)		
Operative Time (min)					
Unilateral Min. – Max. Mean ± SD.		(*n* = 108) 15.0–30.0 21.17 ± 5.42	(*n* = 58) 30.0–50.0 37.61 ± 5.79	U = 5609	< 0.001
Bilateral Min. – Max. Mean ± SD.		(*n* = 34) 30.0–45.0 37.32 ± 4.44	(*n* = 8) 55.0–65.0 57.41 ± 3.81	U = 264	< 0.001
Complications					
Intraoperative bleeding	2 (0.96%)	1 (0.7%)	1 (1.5%)	Fisher’s Exact	1.000
Conversion to open	0	0	0	–	–
Inguinal edema	10 (4.8%)	2 (1.4%)	8 (12.1%)	Fisher’s Exact	0.002
Recurrence	0	0	0	–	–

*SD* Standard Deviation, *U* Mann-Whitney Test, *χ²* Chi-square Test,*MC* Monte Carlo simulation, – = not applicable.

## Discussion

Over the past three decades, the evolution of the laparoscopic management of CIH represents a significant advancement in surgical techniques [3]. Initial applications were exclusively diagnostic for the identification of contralateral PPV during unilateral hernia repairs [5]. The transition to therapeutic application began cautiously, initially limited to female patients to avoid potential iatrogenic injury to the delicate spermatic cord structures in males [5, 10]. With advancements in surgical expertise and instrumentation, the approach was gradually extended to male patients [4]. However, early experiences with the laparoscopic technique revealed certain drawbacks compared to traditional open repair, including higher recurrence rates, the need for multiple incisions, and longer operative durations [11–13].

To address the issue of multiple incisions, several modifications were introduced. For example, Patkowski et al. developed the percutaneous internal ring suturing (PIRS) technique, eliminating the need for multiple ports [6]. Also, introducing smaller laparoscopic instruments and portless approaches further minimized the scar size, enhancing the cosmetic outcomes [14, 15]. Similarly, the challenge of longer operative times was mitigated as surgeons gained experience and refined their techniques, making laparoscopy comparable to or even faster than open surgery in many series [14].

The higher recurrence rates in early reports were attributed to closing the intact peritoneum, particularly in male patients, due to the difficulty of achieving a secure closure over the vas deferens and testicular vessels without leaving a “skip area.” [8, 16]. This limitation prompted the development of techniques that intentionally disrupted the peritoneum to promote scarring for more durable healing at the hernial orifice. Becmeur et al. pioneered the approach of sac disconnection with subsequent peritoneal closure, ensuring secure closure of the peritoneal defect [8]. Similarly, Ostlie and Ponsky modified the extracorporeal approach to include peritoneal cauterization, skipping the area over the vas and vessels before closure of the IR [7].

These advancements significantly reduced recurrence rates in both intracorporeal and percutaneous techniques, dropping from 3.7% in cases with intact peritoneal closure to nearly 0–1% in series where the peritoneum was transected [7, 17]. This can be explained by the distinct regenerative capabilities of peritoneum. In contrast to cutaneous wound healing, which proceeds primarily through epithelial migration from wound margins, peritoneal tissue regenerates intrinsically and forms fewer adhesions when left unsutured [18–20]. This principle was demonstrated in a laparoscopic rabbit model, where induced peritoneal lesions left unsutured remained intact under insufflation pressures up to 35 mmHg while suture-only closures of intact peritoneum showed significantly higher mechanical failure rates under similar conditions. These results indicate that intentional peritoneal injury elicits a more durable healing response than suturing alone [21].

However, some authors have proposed that peritoneal closure may not be necessary, and sac disconnection alone may suffice, especially in cases with small IRs, even in open surgery [9, 22]. They argue that peritoneal closure, although not routinely necessary, requires advanced laparoscopic skills, increases operative time, with potential risk of injury to the vas deferens and testicular vessels [9, 23]. This perspective is supported by the observation that, in laparoscopic orchidopexy, the PPV is often disconnected but not closed, yet hernias rarely develop postoperatively [16, 24].

A new milestone was achieved when Riquelme et al. (2010) introduced the concept of “no ligation, just resection,” reporting a 0% recurrence rate over a four-year follow-up period using sac disconnection alone. However, they selectively repaired IRs with diameters larger than 10 mm [9]. Following this, multiple studies supported the safety and efficacy of sutureless repair. García-Hernández et al. (2012) applied sac disconnection regardless of IRD and reported a low recurrence rate of 0.53%, with complete sac excision down to the testis in scrotal hernias [23]. Another series in 2013 demonstrated a 0% recurrence using sac disconnection alone, with closure reserved for IRs exceeding 20 mm [25]. Similarly, Pant et al. (2014) compared sac ligation versus non-ligation and found that disconnection alone was not only simpler and faster but also had no significant difference in recurrence rates [26]. Novotny et al. (2017) reported no recurrences in girls undergoing laparoscopic sutureless repair irrespective of IRD [27].

In 2018, a stratified algorithm was proposed based on IRD, recommending sac disconnection for 4–15 mm, purse-string suturing for 15–25 mm, and muscular arch repair for defects > 25 mm or in recurrent cases. This approach significantly reduced recurrence rates from 3.7 to 1.2% [28]. Further evidence came from Galván Montaño (2018), Marte (2019), and Bukowski et al. (2020), all of whom reported no recurrences using sutureless repair across all IRD ranges [29–31]. However, Elbatarny et al. (2020) observed higher recurrence rates with disconnection alone in IRDs > 10 mm [17], and Elsayem et al. (2022) reported that while sutureless repair was effective for IRDs < 10 mm, it had a threefold higher recurrence rate than purse-string closure in IRDs between 10 and 15 mm [32]. Most recently, Shalaby et al. (2023) reported no recurrences using a needlescopic sutureless technique for IRDs < 15 mm [33]. In the present study, a tailored surgical strategy was implemented, utilizing an IRD threshold of 10 mm to guide management. This approach resulted in no recurrences in either group at the one-year follow-up.

These findings collectively reinforce the importance of tailored surgical strategies based on IRD and defect size. While sac disconnection and sutureless repair are safe and effective for smaller hernias, larger defects require additional measures, such as purse-string sutures or muscular arch repair, to achieve durable outcomes.

Historically, numerous classification systems for adult inguinal hernias have been developed to stratify hernias based on defect size and complexity, acknowledging the limitations of a “one-size-fits-all” approach [34, 35]. Similarly, pediatric hernias demonstrate considerable variability, influenced by factors such as age, gender, IRD, pelvic depth, and local muscle conditions, necessitating individualized management strategies to optimize outcomes [25, 36]. Although a prior report by Shehata et al. [37] proposed a novel CIH classification adapted from the adult Nyhus system, it was applied in the context of open repair; however, the same scoring system remains applicable to laparoscopic techniques.

Although our study implements the same principle as Riquelme et al., there are three key differences between the two techniques. First, they advocated for complete sac resection, including the parietal peritoneum surrounding IR. This extensive dissection within the inguinal canal, as required for complete sac resection, may increase the risk of injury to the testicular vessels or vas deferens and could lead to postoperative complications such as scrotal edema and hematoma [38]. A study by Endo et al. found that edema rates correlate with the extent of dissection [39]. In contrast, our technique focuses on complete sac disconnection without resection. This approach simplifies the procedure, reduces OT, and minimizes the risk of complications while still achieving a durable repair.

Second, they employed a purse-string suture to close the IR in cases where the IRD exceeded 10 mm. This technique can be challenging and may increase the risk of injuring the vas deferens or testicular vessels if not performed carefully. In our study, we opted for IPT using one or two interrupted sutures. IPTR has been employed for decades in the open repair of adult inguinal hernias. More recently, its laparoscopic adaptation has been reported in the management of CIH, particularly in recurrent cases [40–44]. Our findings, supported by previous studies, suggest that laparoscopic IPTR offers effective and controlled IR repair, minimizing the risk of compression or injury to the vas deferens and testicular vessels.

A potential concern with this approach is postoperative pain due to suture tension, especially given the challenges of pain assessment in pediatric patients. However, comparative studies between IPTR and high ligation have shown no significant differences in postoperative pain levels [40, 42, 43]. Careful suture placement is essential to avoid injury to the femoral branch of the genitofemoral nerve. Anatomical studies show this nerve courses caudally to the inguinal ligament in 84.0% of cases and medially toward the anterior superior iliac spine in 5.2% [45]. To mitigate this risk, sutures should be placed as close as possible to the IR [40].

The third difference is that we utilized a calibrated Maryland dissector to standardize the intraoperative measurement of the IR. This novel technique is simple, quick, and reduces the subjectivity inherent in visual estimation techniques described in earlier studies [9, 25]. Unlike approaches that involve using a thread measured externally on a ruler, our technique provides real-time practicality without the need for additional tools [17, 28]. One study employed US for IRD assessment and found no significant correlation between age and ring diameter [32]. Another group reported that US measurements were significantly smaller than those obtained laparoscopically, attributing the difference to pneumoperitoneum-induced pressure during laparoscopy. They concluded that preoperative US should not guide surgical planning, as intraoperative laparoscopic assessment offers a more accurate representation of IRD [17]. Although our method enhances reproducibility, its accuracy relies on maintaining perpendicular alignment during measurement, a limitation common to other intraoperative tools [46]. Future research could explore the use of laparoscopic calipers or imaging software to further refine this process.

The demographic characteristics of our study population, including age, sex, and hernia laterality, align with findings from previous studies on CIH. The mean age of patients in our study was 5.89 ± 4.05 years in Group A and 5.73 ± 3.91 years in Group B, consistent with the age range reported in the literature [3]. Neonates under 1 month of age were excluded from the study to mitigate the physiological risks associated with laparoscopic surgery in this vulnerable population [47]. This exclusion criterion is consistent with the methodologies of several prior studies [9, 17, 23, 48].

The use of portless instruments with small stab incisions contributed significantly to the minimally invasive nature of the laparoscopic repair. It has been shown to reduce postoperative pain and improve recovery times, as smaller incisions result in less tissue damage and inflammation [43]. Furthermore, the absence of trocars eliminates the risk of port-site hernias, a rare but potential complication associated with traditional laparoscopic techniques [46]. The cosmetic benefits of portless instruments are particularly significant in pediatric patients, as smaller incisions lead to nearly invisible scars, which is a major advantage for both parents and children [29].

In our study, synchronous contralateral PPV was detected intraoperatively in 5.3% of cases, which falls below the 10–20% incidence reported in some studies [3] but aligns with other published series [9]. This variability may be attributed to differences in patient populations and diagnostic criteria. Notably, all detected contralateral PPV in our study were managed successfully with peritoneal disconnection alone, supporting the efficacy of this technique for smaller defects [25].

The OT in our study varied significantly between the two groups, with Group B (IRD > 1 cm) requiring longer procedures due to the additional step of IPTR. The mean OT for unilateral cases in Group A was 21.17 ± 5.42 min, compared to 37.61 ± 5.79 min in Group B. These findings are consistent with previous studies, which have reported longer OT for more complex repairs involving larger IRs [7]. However, our operative times are shorter than those reported in early laparoscopic series, reflecting improvements in surgical techniques and instrumentation [2].

The selection of suture material can significantly influence surgical outcomes [49]. In our study, we employed Vicryl sutures. Some other studies have also utilized Vicryl sutures [8, 26, 44, 48], while others have chosen nonabsorbable options such as Polyester and V-Loc [9, 50, 51]. The rationale for using absorbable sutures stems from the biological healing process following deperitonealization. After exposing the raw muscular arch, wound repair occurs primarily through scar tissue formation rather than dependence on permanent foreign material retention [8, 9]. Unlike nonabsorbable sutures like Polyester or V-Loc, which remain permanently in the tissue, absorbable sutures provide temporary support during the critical healing phase and are gradually absorbed, minimizing the risk of long-term complications such as chronic pain, suture migration, or granuloma formation [52].

Intraoperative complications were rare in our study, with only two cases (0.96%) of minor periperitoneal bleeding, both of which stopped spontaneously. This low complication rate is comparable to or better than those reported in other laparoscopic series [9, 25]. Transient inguinal edema was observed in 1.4% of Group A and 12.1% of Group B, with all cases resolving spontaneously within two weeks. The higher incidence of edema in Group B is likely attributable to the additional manipulation and suture placement required for IR narrowing [39]. These findings are consistent with previous studies, which have reported edema rates ranging from 5 to 15% following laparoscopic repair, particularly in cases involving larger internal rings [28]. The absence of vas deferens or testicular vessel injuries, as well as persistent edema or hematoma, highlights the safety of our technique. Utilizing hook monopolar diathermy in cutting mode enabled precise peritoneal disconnection with minimal lateral thermal damage [39]. By creating small peritoneal rents and gently sweeping the vas and vessels away without direct diathermy contact, we ensured complete sac disconnection while preserving adjacent structures. The lateral damage caused by cutting current is much less than that caused by coagulation current.

Postoperative follow-up compliance was high (95% at one year), thereby enabling reliable ascertainment of both early and late outcomes. Given the introduction of a novel technique, an extended follow-up protocol was intentionally implemented to ensure thorough surveillance for potential complications. Attrition was minimal (5% at one year), well within accepted standards for prospective clinical studies, and was unlikely to have introduced bias into the findings, as no complications or recurrences were observed beyond 6 months among patients who completed follow-up [53]. Comparative data on one-year retention in the literature remain limited, as most published series reported recurrence outcomes without explicitly documenting long-term follow-up completion rates [9, 14, 28, 54]. Table 2..

**Table 2.** Comparative analysis of published studies utilizing laparoscopic sac disconnection only for CIH

Study	Year	Number of hernias	Males	Females	Age Range	Technique	IRD (mm)	Follow-Up Duration	Recurrence Rate
Riquelme et al. (9)	2010	91	76	15	2 m to 11 y	Sac disconnection and complete removal	< 10	4 years	0%
García-Hernández et al. (23)	2012	285	233	53	1 m to 15 y	Sac disconnection and complete removal	All IRDs	55 months	0.53%
Shehata et al. (25)	2013	78	42	14	6 m to 15 y	Sac disconnection	< 20	2 years	0%
Pant et al. (26)	2014	38	Not specified	Not specified	12 m to 12 y	Sac disconnection	All IRDs	6 months	5.3%
Novotny et al. (27)	2017	80	0	67	1 m to 16 y	Sac disconnection	All IRDs	25 months	0%
Shehata et al. (phase II)(28)	2018	80	Not specified	Not specified	3 m to 15 y	Sac disconnection	< 15	15.5 months	1.2% (for the entire cohort, not just sutureless repairs)
Galván Montaño (29)	2018	26	14	12	3 m to 13 y	Sac disconnection	All IRDs	14 months	0%
Marte (30)	2019	28	16	12	3 m to 7 y	Sac disconnection	< 15	18 months	0%
Bukowski et al. (31)	2020	67	67	0	3 m to 10 y	Sac disconnection	All IRDs	6 months	0%
Elbatarny et al. (17)	2020	20	12	3	1 m to 2 y	Sac disconnection	All IRDs	12 months	15% (cases with IRD > 10 mm)
Elsayem et al. (32)	2022	82	Not specified	Not specified	2 m to 8 y	Sac disconnection	All IRDs	12 months	3.3% (cases with IRD > 10 mm)
Shalaby et al. (33)	2023	128	70	44	4.62 ± 2.14 y	Sac disconnection	< 15	12 months	0%
Present Study	2025	142	117	25	3 m to 12 y	Sac disconnection	< 10	1 year	0%

*“not specified” indicates that the study did not provide detailed data for that category. IRD* Internal Ring Diameter

## Conclusion

Laparoscopic hernial sac disconnection without peritoneal closure represents a safe and effective treatment for CIH with small internal rings (< 1 cm). Avoiding peritoneal closure can reduce operative time and minimize possible injury to the vas deferens and vascular structures during suturing.

For defects larger than 1 cm, laparoscopic disconnection with repair of the muscle arch, while still avoiding peritoneal closure, is recommended to prevent hernia recurrence. However, this leads to a longer operative time and a slight increase in minor self-limiting complications.

This tailored approach can significantly reduce recurrence rates while obviating unnecessary steps that can cause morbidity. Further studies with long-term follow-up are therefore recommended.

## Supplementary Information

Below is the link to the electronic supplementary material.

Supplementary Material 1 (DOCX 24.9 KB)

## Data Availability

The datasets used and/or analysed during the current study are available from the corresponding author on reasonable request.

## Abbreviations

CIH: Congenital Inguinal Hernia
IPTR: Iliopubic Tract Repair
IPT: Iliopubic Tract
IRD: Internal Ring Diameter
IR: Internal Ring
OT: Operative Time
PPV: Patent Processus Vaginalis
PIRS: Percutaneous Internal Ring Suturing
SD: Standard Deviation
TATA: Transverse Arch of Fascia Transversalis
US: Ultrasound
